# Effects of 60 Hz magnetic fields on teenagers and adults

**DOI:** 10.1186/1476-069X-12-42

**Published:** 2013-05-24

**Authors:** Sung Kean Kim, Jae Lim Choi, Min Kyung Kwon, Joon Yul Choi, Deok Won Kim

**Affiliations:** 1 Department of Medical Engineering, Yonsei University College of Medicine, Seoul, Republic of Korea; 2 Graduate Program in Biomedical Engineering, Yonsei University, Seoul, Republic of Korea; 3 LS Industry Systems Co., Ltd, Chunan, Choongnam, Republic of Korea; 4 Brain Korea 21 Project for Medical Science, Yonsei University College of Medicine, Seoul, Republic of Korea

**Keywords:** ELF, Physiological changes, Subjective symptoms, Perception

## Abstract

**Background:**

As use of electrical devices has increased, social concerns about the possible effects of 60 Hz electromagnetic fields on human health have increased. Accordingly, the number of people who complain of various symptoms such as headache and insomnia has risen. Many previous studies of the effects of extremely low frequency (ELF) magnetic field exposure on children have focused on the occurrence of childhood leukaemia and central nervous system cancers. However, very few provocation studies have examined the health effects of ELF magnetic fields on teenagers.

**Methods:**

In this double-blind study, we simultaneously investigated physiological changes (heart rate, respiration rate, and heart rate variability), subjective symptoms, and magnetic field perception to determine the reliable effects of 60 Hz 12.5 μT magnetic fields on teenagers. Two volunteer groups of 30 adults and 30 teenagers were tested with exposure to sham and real magnetic fields for 32 min.

**Results:**

ELF magnetic field exposure did not have any effects on the physiological parameters or eight subjective symptoms in either group. Neither group correctly perceived the magnetic fields.

**Conclusions:**

Physiological data were analysed, subjective symptoms surveyed, and the percentages of those who believed they were being exposed were measured. No effects were observed in adults or teenagers resulting from 32 min of 60 Hz 12.5 μT magnetic field exposure.

## Background

As the use of electrical devices increases, social concerns about the biological effects of electromagnetic fields (EMF) on human health are growing. In our daily lives, 50 or 60 Hz is the most common frequency of electricity, which falls in the extremely low frequency (ELF) range. Particularly, concerns have been expressed about the potential adverse effects of EMF exposure on developing children. The World Health Organization (WHO) Research Agenda for ELF fields concluded that there were no substantive health issues related to ELF electric fields at levels generally encountered by members of the public. The WHO Research Agenda recommended further research concerning the possible effects of exposure to ELF magnetic fields [1]. However, most previous studies of ELF magnetic field exposure on children have focused on the occurrence of childhood leukemia and central nervous system cancers, the malignancies most frequently mentioned in connection with ELF-EMF among children [2]. Several pooled analyses and reviews found a generally consistent, albeit moderate, association between exposure of children to EMFs and the risk of childhood leukaemia [3-5], although no definitive biological mechanism has been identified [6].

The autonomic nervous system (ANS) plays an important role not only in physiological situations, but also in various pathological settings. Among the different available noninvasive techniques for assessing the ANS, heart rate variability (HRV), which is obtained from heart rate, has emerged as a simple, noninvasive method to evaluate the sympathovagal balance at the sinoartrial level [7]. Respiration rate is also closely associated with HRV [8]. Therefore, we selected the three parameters including heart rate, HRV, and respiration rate to assess ANS activity. There are some studies investigating the effects of ELF-EMFs on heart rate and HRV only for adults [9-11].

Some previous studies investigated subjective symptoms or EMF perception in ELF-EMFs for adults [12-14]. However, to our knowledge, there are very few studies regarding subjective symptoms and EMF perception in children. We examined subjective symptoms and EMF perception as well as physiological parameters. Therefore, when the three factors above are measured simultaneously, the results could be more reliable.

Children might be more sensitive to radiation in some or all parts of the electromagnetic spectrum. Concerns about the potential vulnerability of children to EMFs have been raised because of the potentially greater susceptibility of their developing nervous systems. In addition, their brain tissue is more conductive, EMF penetration is greater relative to their head size, and they will have a longer lifetime of exposure than adults [15].

In this double-blind study, we measured heart and respiration rates for both adult and teenager groups, then obtained HRV using the measured heart rate. In addition, participants were asked to describe subjective symptoms and EMF perception during pre-exposure, sham and real exposures, and post-exposure. The aim of this study was to test whether 60 Hz magnetic fields affect heart rate, respiration rate, and HRV, or give rise to subjective symptoms in adults and teenagers. We also compared the ability of adults and teenagers to perceive exposure to a magnetic field. We tested the null hypothesis that we would observe no differences in autonomic nervous system, subjective symptoms, or EMF perception between real and sham exposures for the two groups.

## Methods

### Subjects

Participants with electromagnetic hypersensitivity (EHS) who attributed their symptoms to appliances and/or high voltage transmission lines or mobile phones were excluded using the EHS screening tool developed by Eltiti et al. [16]. In addition, only healthy subjects with no disease or subjective symptoms who were not on medications were included. This double-blind study had 60 participants in two groups: 30 adults and 30 teenagers (because the experiment was demanding and potentially stressful, we did not recruit children younger than 13 years old). As shown in Table 1, no significant differences were seen in male-to-female ratio, height, weight, body mass index, smoking status, or TV viewing time per day between the two groups. However, significant differences were seen in age, computer usage per day, and mobile phone usage period between the two groups.

**Table 1:** Demographics of participants

	**Adult**	**Teenager**	***P*-value**
No. of subjects (n)	30	30	-
Male: female	15 : 15	14 : 16	0.796
Age (yr)	27.9 ± 5.9	14.8 ± 1.4	0.000
Height (cm)	166.4 ± 7.2	166.4 ± 6.9	0.985
Weight (kg)	58.4 ± 7.7	57.0 ± 10.6	0.304
body mass index (kg/m^2^)	21.0 ± 1.8	20.5 ± 2.8	0.110
Non-smoker: smoker	25 : 5	26 : 4	1.000
Computer usage time (h/d)	6.5 ± 3.9	2.3 ± 1.7	0.000
TV viewing time (h/d)	1.4 ± 1.6	1.9 ± 1.8	0.068
Mobile phone usage periods (yr)	9.9 ± 2.9	4.5 ± 1.9	0.000

Participants were advised not to consume caffeine, smoke, or exercise, and to get sufficient sleep before the day of the experiment, to minimise confounding factors. All subjects were recruited by advertisements at the Yonsei University Health System in Seoul, Korea, were informed of the purpose and procedure of the experiment, and were required to give written consent to participate. The Institutional Review Board of the Yonsei University Health System approved the protocol of this study (project number: 1-2010-0031).

### Experimental setup

The laboratory was used exclusively for this experiment, and all electrical devices were unplugged except for our instruments, to minimise background field levels. Background ELF fields in the laboratory were measured at head level to ensure that they did not influence the participants. The average ELF electric and magnetic fields were 0.8 ± 0.0 V/m and 0.03 ± 0.00 μT, measured using an electric and magnetic field analyser (EHP-50C, NARDA-STS, Milan, Italy).

**Figure 1:**
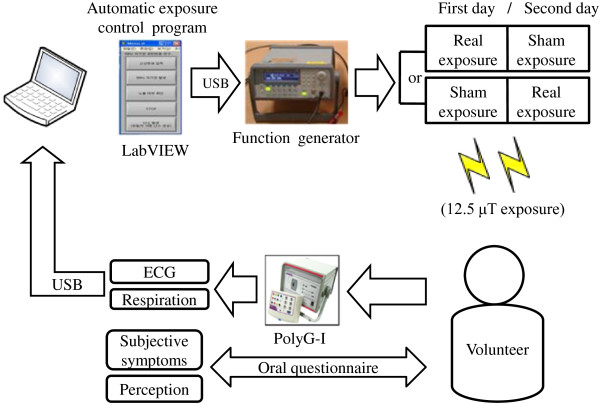
Experimental configuration of the 60 Hz magnetic field exposure system.

**Figure 2:**
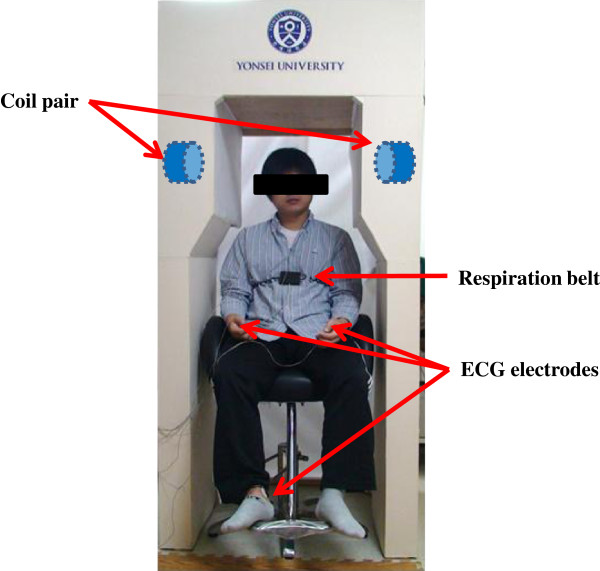
Photo of experimental setup.

Figure 1 shows the experimental setup used to examine participants and the 60 Hz magnetic field exposure system. The magnetic field generator consisted of an arbitrary function generator (33220A, Agilent, Santa Clara, CA) and a coil pair constructed for this study. The coil pair was adopted to produce more uniform magnetic fields in a head than a single coil. Each coil had 2000 turns with a radius of 10 cm, height of 20 cm, and coil thickness of 0.7 mm. The output of the function generator was controlled using LabVIEW 2009 (National Instruments, Austin, TX). During the experiment, participants were positioned in the centre of the space between the coils (Figure 2). The distance between the coils was 50 cm and the coil pair was covered with fabric to conceal it. The participant’s head was positioned in the centre between the coils by adjusting the chair height to expose the head at 12.5 μT. Since the brain controls the ANS, many previous studies exposed the head to magnetic fields [11,17,18]. We selected 12.5 μT because this was the strongest magnetic field measured directly under most transmission lines in Republic of Korea according to Korea Electric Power.

### Experimental procedures

No information was given to participants except that they would be asked about symptoms and EMF perception at the beginning of the first experimental day. Sham and real sessions were conducted as a double-blind test to minimise test bias from a participant and an experimenter recognising the operational state of the magnetic field generator. Experiments were performed for two days, one day for a real session and a second day for a sham session (or vice versa). Regardless of whether the sham or real exposure came first, the second session was always conducted at approximately the same time of the day as the first session, to maintain the participant’s physiological rhythm. The order of sham and real sessions was randomly assigned to each subject and counterbalanced on our automatic exposure control program using LabVIEW 2009 (National Instruments) to minimise experimental bias. The sham exposure was the first session for 17 teenagers and 18 adults. Time between the sessions was a minimum of one day and a maximum of 20 days.

The average ELF electric and magnetic fields were 0.75 ± 0.10 V/m and 0.03 ± 0.00 μT during sham exposure and 3.52 ± 0.95 V/m and 12.49 ± 0.02 μT during real exposure, respectively. Room temperature and relative humidity, which could considerably affect outcomes, were recorded and maintained as shown in Table 2. For the adult group, room temperature showed no significant differences between real and sham sessions (*P* = 0.893). Humidity also showed no significant differences between real and sham sessions (*P* = 0.708). For the teenager group, room temperature showed no significant differences between real and sham sessions (*P* = 1.000). Humidity also showed no significant differences between real and sham sessions (*P* = 0.155). For the sham sessions, room temperature showed no significant differences between adult and teenager groups (*P* = 0.792). Humidity also showed no significant differences between adult and teenager groups (*P* = 0.871). For the real sessions, room temperature showed no significant differences between adult and teenager groups (*P* = 0.896). Humidity also showed no significant differences between adult and teenager groups (*P* = 0.524).

**Table 2:** Room temperature (°C) and relative humidity (%) in the real and sham sessions for the adult and teenager groups (mean ± SD (min-max))

	**Group**	**Real**	**Sham**	***P*-value**
Temperature	Adult	24.1 ± 0.9 (22–26)	24.1 ± 1.1 (22–27)	0.893
	Teenager	24.2 ± 1.1 (22–26)	24.2 ± 0.9 (22–26)	1.000
Humidity	Adult	41.2 ± 6.2 (33–55)	41.0 ± 6.4 (30–55)	0.708
	Teenager	40.4 ± 2.2 (35–45)	40.8 ± 2.0 (37–45)	0.155

### Physiological measurements

**Figure 3:**
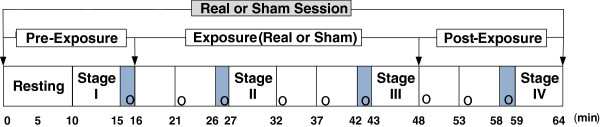
**Experimental procedures for measuring physiological changes and investigating symptoms and perception.** Four shaded areas are periods in which participants were questioned about eight symptoms. “o” indicates inquiry about EMF perception during each session.

The duration of each session was 64 min, as shown in Figure 3. Before experiments, participants were instructed to rest in a sitting position for at least 10 min. Physiological data were collected for 5 min for each of four different stages: pre-exposure (stage I), after 11 min of exposure (stage II), after 27 min of exposure (stage III), and post-exposure (stage IV) [19]. At each stage, ECG and respiration were simultaneously measured for 5 min because of the minimum data requirement for HRV [20]. Heart rate, respiration rate, and HRV were obtained with a computerised polygraph (PolyG-I, Laxtha, Daejeon, Korea) with a sampling frequency of 512 Hz. Data were transferred to a laptop computer (X-note R500, LG Electronics, Seoul, Korea) and analysed using data acquisition software (Telescan 0.9, Laxtha) and analysis software (Complexity software, Laxtha). ECG was recorded through Ag-AgCl electrodes (2223, 3M, St. Paul, MN) placed on both arms and the right leg of participants using the PolyG-I.

We first obtained heart rate from ECGs and then acquired HRV and the power spectrum of HRV. High-frequency power (HFP) reflects effects on respiratory sinus arrhythmia, an index of parasympathetic nerve activity, whereas low-frequency power (LFP) reflects effects on both sympathetic and parasympathetic nerves [21]. In this study, the LFP/HFP ratio was used as an index of autonomic nerve activity balance. Respiratory inductance plethysmography, with an excitation frequency of 3 MHz, was used to measure respiration rate. Subjects wore a coiled band around their upper abdomen for measurement of inductance changes resulting from cross-sectional change, as shown in Figure 2.

### Subjective symptoms and perception of EMF

The four shaded areas in Figure 3 denote periods during which subjects were questioned about eight symptoms, with each period lasting approximately 1 min. The eight subjective symptoms of throbbing, itching, warmth, fatigue, headache, dizziness, nausea, and palpitation were evaluated through verbal surveys, which were graded on a 4-point scale ranging from 1 (no sensation) to 4 (strong sensation) as suggested by Koivisto et al. [22]. In addition, perception of EMF exposure was investigated every 5 min throughout the entire session, denoted by an “o” in Figure 3[23]. Subjects were asked to answer the question “Do you believe that you are exposed right now?” nine times during each session. Percentages of those who believed they were being exposed were calculated for pre-exposure, exposure, and post-exposure periods. The total number of inquiries was 300 (5 × 60) during actual exposure and 780 (13 × 60) during non-exposure; the total number of subjects was 60 (30 + 30).

### Data analysis

A repeated two-way analysis of variance (ANOVA) was performed using SPSS software (SPSS 18, SPSS, Chicago, IL) to investigate differences in heart rate, respiration rate, and relative change in LFP/HFP with exposure and stage for adult and teenager groups. *P* < 0.05 was considered statistically significant. Subjective symptoms, which are ordered paired data, were analysed using a nonparametric Wilcoxon signed-rank test. A total of 64 *P*-values (4 stages × 8 symptoms × 2 groups) were obtained for the real and sham exposure sessions for the eight symptoms at four stages in both groups. The significance level was adjusted to 0.0125 (0.05/4) because testing was performed in four stages.

Two exposure sessions took place for each participant with nine perception inquiries for each session, as shown in Figure 3. For each session, one inquiry was during pre-exposure, five inquiries during sham or real exposure, and three inquiries during post-exposure. In both groups, the percentages of those who believed they were being exposed were obtained and evaluated for significant differences between real and sham sessions using McNemar’s test. The pre-exposure period (first inquiry) of the sham sessions was compared with the real sessions to test whether conditions before sham and real exposures were the same. The sham exposure period was compared with the real exposure period to test whether the subjects could detect the fields (second through sixth inquiries). The post-exposure period after sham exposure was compared with the post-exposure period after real exposure to test whether the real exposure influenced exposure perception in the post-exposure period (seventh through ninth inquiries).

**Figure 4:**
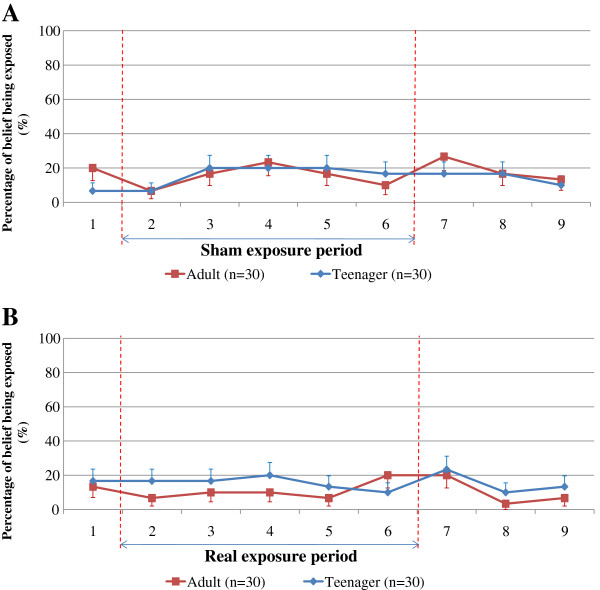
**Percentages who believed they were being exposed at nine inquiry points for adults and teenagers for sham (A) and real (B) exposure sessions.** Bars indicate standard errors.

The significance level of the exposure period was adjusted to 0.01 (0.05/5) and significance for the post-exposure period was adjusted to 0.017 (0.05/3) because testing was for five and three inquiries. A chi-square test was applied to evaluate differences in the percentages of those who believed they were being exposed between the adult and teenager groups for sham and real exposure sessions, as shown in Figure 4. Fisher’s exact test was used for the same analysis when the expected values in any cells in the contingency table were below 5.

## Results

### Adult and teenager groups

We screened 32 teenagers and excluded two because they did not show up on the second experimental day. All adults attended the second day after attending the first day. No participants discontinued during the experiment for both groups.

### Physiological variables

Heart rate, respiration rate, and LFP/HFP ratios of the adult and teenager groups during real and sham exposures are in the top of Table 3. For analysis of relative changes in LFP/HFP, LFP/HFP values for real and sham were expressed relative to the corresponding values of stage I at the pre-exposure period (defined as 100%) because of large individual variation. A repeated two-way ANOVA showed no significant differences in heart rate or respiration rate for stage or exposure in either group. However, LFP/HFP showed significant differences by stage in both groups, as shown in the bottom of Table 3. Therefore, a Bonferroni post hoc test was done after two-way ANOVA to investigate differences in LFP/HFP between stages for each group. For the adult group, LFP/HFP showed no significant difference between real and sham exposures (*P* = 0.883), but did show a significant difference among stages (*P* < 0.001). For the teenager group, LFP/HFP was not significantly different between real and sham exposures (*P* = 0.965), but was significantly different among stages (*P* < 0.001).

**Table 3:** Descriptive and statistical tests for heart rate, respiration rate, and LFP/HFP (%) among stage, exposure, and interaction

	**Heart rate (bpm)**				**Respiration rate (bpm)**				**LFP/HFP (%)**			
	**Adult**		**Teenager**		**Adult**		**Teenager**		**Adult**		**Teenager**	
	**Sham**	**Real**	**Sham**	**Real**	**Sham**	**Real**	**Sham**	**Real**	**Sham**	**Real**	**Sham**	**Real**
Stage: mean (standard error)												
I	78.7 (2.1)	78.2 (2.1)	81.9 (1.4)	80.9 (1.8)	18.4 (0.4)	18.4 (0.5)	17.9 (0.5)	18.6 (0.5)	100.0 (0.0)	100.0 (0.0)	100.0 (0.0)	100.0 (0.0)
II	78.0 (2.1)	76.6 (1.9)	82.0 (1.4)	80.8 (1.6)	18.9 (0.4)	18.2 (0.4)	18.1 (0.5)	18.2 (0.4)	144.9 (11.9)	156.3 (19.5)	129.3 (15.3)	142.4 (16.7)
III	76.7 (2.1)	77.0 (2.0)	80.7 (1.3)	80.9 (1.4)	18.6 (0.4)	18.5 (0.4)	18.0 (0.5)	18.4 (0.4)	148.0 (16.5)	175.8 (21.2)	156.5 (20.4)	166.5 (22.9)
IV	78.3 (2.1)	76.9 (1.9)	81.2 (1.4)	80.6 (1.4)	19.2 (0.4)	18.5 (0.4)	18.5 (0.5)	18.6 (0.4)	178.0 (21.8)	148.2 (9.9)	177.0 (22.8)	157.0 (22.9)
Factor (*P*-value)												
Exposure	0.623		0.487		0.115		0.135		0.883		0.965	
Stage	0.075		0.669		0.130		0.165		< 0.001*		< 0.001*	
Interaction (exposure and stage)	0.318		0.376		0.323		0.102		0.180		0.570	

* *P* < 0.05, bpm; beats per min.

LFP/HFP; low-frequency power/high-frequency power (power spectrum of heart rate variability).

Stage I; pre-exposure, Stage II; after 11 min of exposure, Stage III; after 27 min of exposure, Stage IV; post-exposure.

### Subjective symptoms

Neither the adult nor the teenager group showed significant differences in any of the eight subjective symptoms surveyed (throbbing, itching, warmth, fatigue, headache, dizziness, nausea, and palpitation) between sham and real sessions at any of the four stages.

### Percentage of belief of being exposed

Table 4 shows the percentages of subjects who believed they were being exposed during exposure (real or sham) in the adult and teenager groups. We compared the percentages of those perceiving exposure during actual exposure (second through sixth inquiries) using McNemar’s test and found no significant difference between real and sham exposures in the adult or teenager groups. To test for delayed effects of real exposure on post-exposure perception (seventh through ninth inquiries), we applied the same test and found no significant difference in the percentages of those who believed they were being exposed following real and sham exposures in the adult (*P* = 0.687, *P* = 0.125, *P* = 0.625) or teenager (*P* = 0.687, *P* = 0.625, *P* = 1.000) groups. Also, no significant difference was seen during pre-exposure (first inquiry) between real and sham exposures in adult (*P* = 0.687) or teenager (*P* = 0.250) groups, indicating that the conditions experienced by participants before real and sham exposures were the same. Similarly, a chi-square test for trend showed that the percentages of those who believed they were being exposed during pre-exposure, sham exposure, and post-exposure were not significantly different in the adult (*P* = 0.850) or teenager (*P* = 0.508) groups. This demonstrated that conditions could not be distinguished for participants throughout sham-exposure sessions.

**Table 4:** **Percentages of adults and teenagers who believed they were being exposed during exposure and *P *values for sham and real exposures**

**Group**	**Session**	**Exposure**									
		**2nd**		**3rd**		**4th**		**5th**		**6th**	
		**Mean (%)**	***P*-value**	**Mean (%)**	***P*-value**	**Mean (%)**	***P*-value**	**Mean (%)**	***P*-value**	**Mean (%)**	***P*-value**
Adult (n = 30)	Sham	6.7	1.000	16.7	0.625	23.3	0.219	16.7	0.250	10.0	0.375
	Real	6.7		10.0		10.0		6.7		20.0	
Teenager (n = 30)	Sham	6.7	0.375	20.0	1.000	20.0	1.000	20.0	0.687	16.7	0.625
	Real	16.7		16.7		20.0		13.3		10.0	

Figure 4 shows the percentages of participants in the adult and teenager groups for each inquiry number who believed they were being exposed in sham (Figure 4A) and real (Figure 4B) exposure sessions. No significant differences were seen between the adult and teenager groups in all inquiries during sham or real exposure session. Even though both groups showed low percentages of belief of being exposed during the sham exposure period (Figure 4A), they also showed low percentages during the real exposure period (Figure 4B). Therefore, we concluded that neither the adult nor the teenager group correctly perceived the magnetic fields.

## Discussion

Obtaining approval of parents was difficult, which made recruitment of teenagers difficult. Another limitation of this study was that we used only one exposure intensity. In future studies, the effects of ELF-EMFs in various exposure intensities on adults and teenagers should be investigated. Although the order of sham and real sessions was randomly assigned to each subject and counterbalanced on our automatic exposure control program to minimise experimental bias, more subjects received sham exposure for the first session. Ideally, the same number for each session is the best. However, the skewness is small and probably makes no difference.

Neither the adults nor the teenagers showed significant differences in heart or respiration rate between real and sham exposures or among stages. For LFP/HFP, however, significant differences were seen between some stages during both real and sham exposure sessions in both groups. One disadvantage of the LFP/HFP analysis is that it is considerably influenced by stress, which can increase or decrease LFP/HFP [24]. Hjortskov et al. [25] reported that psychological stress could result in an increased LFP/HFP. Nam et al. [26] reported that LFP/HFP monotonically increased at each exposure stage in both EHS and non-EHS groups during 30 min of sham exposure. In this experiment, one of the potential sources of stress was the requirement that the subjects not move during the 64-min experiment. In fact, the “no-movement” requirement was the factor that drew the most complaints from the participants. Therefore, the significant increase in LFP/HFP with time during both the real and sham exposure sessions for both groups must have resulted from factors other than field exposure such as psychological stress, anxiety, or environmental factors.

Sait et al. [9] reported that a 50 Hz 28 μT magnetic field had no effect for either heart rate or HRV in 20 adults. Graham et al. [27] performed ELF-EMF meta-analysis of seven studies and concluded that there was no effect of overnight exposure to 60 Hz magnetic fields on either heart rate or HRV. However, in some studies, exposure to 50/60 Hz magnetic fields ranging from 20 μT to 200 μT have been found to cause slowing of the heart rate and as well as to change HRV [10,11,28]. Laboratory research into the effects of EMF exposure on heart rate and HRV has been inconclusive [29]. To our knowledge, there are very few provocation studies regarding ELF EMFs on children or teenagers.

An interesting pattern was observed in the LFP/HFP results in Table 3. In sham sessions, LFP/HFP continuously increased from stage I to stage IV for the adult and teenager groups. However, in real sessions, LFP/HFP increased more rapidly, but then decreased during stage IV for both groups. This pattern could represent a subtle magnetic field effect that increased LFP/HFP during exposure (stage II and III), but disappeared after exposure (stage IV), which was not observable as statistically significant with the current study design and group size. The interaction term (exposure and stage) was not statistically significant in either the adult or teenager groups. As the pattern of changes was similar in the both groups, we performed a further check and found that the interaction term was not significant in a combined analysis of adult and teenager groups (*P* = 0.103).

In this study, neither the adult group nor the teenager group showed significant differences in any of the four stages between real and sham sessions for any of the eight symptoms surveyed. Mortazavi et al. [30] concluded that no significant differences occurred in the prevalence of symptoms such as headache, fatigue, difficulty in concentration, vertigo, or attention disorders between healthy university students who used and did not use computer monitors (cathode ray tubes or CRTs). These monitors produce ELF as well as very low frequency (VLF) magnetic fields. McMahan et al. [31] also reported that the incidence of subjective symptoms was higher in subjects who were worried about the effects of magnetic fields, rather than in subjects actually exposed in people living near power lines. In conclusion, ELF magnetic fields did not give rise to subjective symptoms in adults or teenagers in this study.

No significant differences were seen in the percentages of participants who believed they were being exposed between the real and sham exposures in either the adult or the teenager group. No significant differences in percentages of perception were seen for either group among participants who believed they were being exposed during either pre-exposure or post-exposure periods between real and sham exposures. Also, no significant differences were observed in the percentages of perception for either the adult or teenager groups during sham exposure sessions (pre-exposure, sham exposure, post-exposure). Therefore, our experimental protocol appeared to be minimally biased since we confirmed no delayed effects, no differences in pre-exposure condition, and no difference in the percentage of those who believed they were being exposed during the pre-exposure, sham exposure, and post-exposure periods.

## Conclusions

In both adults and teenagers, exposure to a 60 Hz magnetic field had no effects on heart rate, respiration rate, LFP/HFP, or subjective symptoms. Neither adults nor teenagers could perceive the magnetic fields, and we observed no indication that the teenagers perceived the magnetic field better than the adults. Therefore, based on our physiological data, survey of subjective symptoms, and percentages of participants who believed they were being exposed, we conclude there are no effects of 32 min exposure to a 60 Hz 12.5 μT magnetic field on the variables examined in adults or teenagers.

## Abbreviations

ANOVA: Analysis of variance; ANS: Autonomic nervous system; CRT: Cathode ray tube; ECG: Electrocardiogram; EEG: Electroencephalograms; EHS: Electromagnetic hypersensitivity; ELF: Extremely low frequency; EMF: Electromagnetic field; HFP: High-frequency power; HRV: Heart rate variability; LFP: Low-frequency power; n: Number; VLF: Very low frequency; WCDMA: Wideband code division multiple access; WHO: World Health Organization.

## Competing interests

The authors declare that they have no competing interests.

## Authors’ contributions

SKK recruited subjects, collected experimental data. JLC performed statistical analyses. MKK and JYC collected experimental data. DWK contributed to the development of the study protocol and editing of the manuscript. All authors read and approved the final manuscript.
